# Risk Factors for Postoperative Complications in Hernia Repair

**DOI:** 10.7759/cureus.51982

**Published:** 2024-01-09

**Authors:** Bandar Saad Assakran, Atheer M Al-Harbi, Hala Abdulrahman Albadrani, Rogayah S Al-Dohaiman

**Affiliations:** 1 General Surgery, King Fahad Specialist Hospital, Buraydah, SAU; 2 College of Medicine, Qassim University, Buraydah, SAU

**Keywords:** open hernia repair, mesh repair, risk factors, hernia repair, post-op complications

## Abstract

Background and objective

Hernias of the abdominal wall were prevalent in people of all ages worldwide, with an overall prevalence of 1.7 percent. Recently, laparoscopic and Lichtenstein mesh repairs have become popular as they provide a rapid return to normal activities with low recurrence rates. There is a relatively high risk of complications following hernia repair, such as wound seroma/hematoma, urinary retention, and superficial incisional infection. As for complications that may develop after hernia repair, we discussed risk factors contributing to postoperative complications after hernia repair in this study.

Methods

This study was a retrospective descriptive study of all patients who underwent hernia repair. The cohort of patients data would be collected from patients using an interview-based questionnaire. The data obtained will be entered into a spreadsheet and analyzed using the Statistical Package for the Social Sciences (SPSS) 23.0 package (IBM Corp., Armonk, NY).

Results

In the current study, we collected data from 274 patients with hernia. The majority of participants were male (79.9%, n = 219), while (37.7%, n = 100) overweight, and (29.8%, n = 79) obese. Among the participants, (82.8%, n = 227) did not experience any complications, while (17.2%, n = 47) reported complications. The types of complications observed were as follows: seroma formation (2.9%, n = 8), wound infection (5.8%, n = 16), and mesh infection (1.8 %, n = 5). There were no significant associations between gender and the presence of complications. However, a significant association was found between BMI and complications (p < 0.001). Diabetes mellitus also showed a significant association with complications (p = 0.005), with a higher proportion of complications among participants with diabetes.

Conclusion

In the current study, we found a significant prevalence of postoperative complications with significant risk factors such as obesity and diabetic mellitus. Additional investigation is warranted to validate these correlations and investigate supplementary variables that could potentially contribute to postoperative problems in hernia surgery.

## Introduction

A part or structure is abnormally protruding through tissues that typically contain it, called a hernia. A hernia is composed of a sac, a neck, and contents. In most cases, abdominal hernias contain fat and bowel. However, any solid or hollow abdominal viscus can be partially or entirely within the hernial sac [1]. Hernias of the abdominal wall were prevalent in people of all ages worldwide, with an overall prevalence of 1.7 percent [2]. A study in Saudi Arabia found that 38.8% of people suffer from abdominal hernias [3]. The prevalence of abdominal hernias was 11.7 in another study from Arar, Saudi Arabia [4].

A hernia in the abdominal wall can be congenital, meaning that the abdominal wall does not close properly or is acquired [5]. Numerous factors and causes are associated with hernias, including pre-existing weakness of abdominal muscles, smoking, prior surgeries, and increased intra-abdominal pressure such as obesity, coughing, straining, heavy lifting, and pregnancy [6,7].

There are many types of hernia, including indirect and direct inguinal hernia, femoral hernia, umbilical hernia, Richter hernia, incisional hernia, and more [5]. Despite that, inguinal hernia, which occurs in the groin area, is the most common type [8]. Even though hernias are not emergencies, they can lead to severe complications, including incarceration, meaning that the contents of the hernia cannot be reduced. Thus, bowel obstructions will result, and blood flow to the hernia will be cut off, leading to strangulations [9,10]. It is common for patients suffering from strangulation to present with a mass in the abdomen that cannot be reduced and localized pain combined with nausea and vomiting [9,11].

Although hernia can be asymptomatic, the patient may complain of a visible bulge, which can cause vague discomfort. However, it can often be reduced manually, even for those that seem large [9]. Usually, abdominal hernia is diagnosed clinically when the patient is in a standing position. As hernias may become apparent at higher abdominal pressure, the patient should cough or perform a Valsalva maneuver when palpating the abdominal wall. On the other hand, clinical diagnosis can be challenging, particularly in obese patients. An abdominal imaging study is used to determine the diagnosis in this case [9,12].

Hernias can be treated using various conservative and surgical approaches, with the choice depending on criteria such as the type and condition of the hernia, as well as the patient's age and history of previous procedures. Two commonly used surgical methods are laparoscopic mesh repair and open mesh repair, both of which require general anesthesia. The laparoscopic mesh repair process involves making incisions in the skin above the umbilicus for the first trocar (10-12 mm), above the pubic symphysis for the second trocar (5 mm), and midway between the first and second trocars for the third trocar (10-12 mm). Upon exposing the inferior epigastric vessels, we proceed to dissect Copper's ligament until it reaches its junction with the femoral vein. Subsequently, we remove the hernia sac and preperitoneal fat from the hernia opening through gentle traction. A mesh is then positioned over the myopectineal orifice and secured in place with staples. Finally, we staple the lower border of Copper's ligament, ensuring that all staples are properly placed and any excess mesh is removed. Assess hemostasis, extract the trocars, and seal the incision using staples or sutures [13].

The open mesh repair procedure begins with making an incision in the marked groin area. This allows the surgeon to expose and cut the external oblique aponeurosis. Next, the surgeon locates and protects the ilioinguinal nerve. Then, the surgeon gently encircles the spermatic cord (or round ligament in females) at the external ring using a penrose drain. By dissecting free from surrounding structures, the surgeon is able to open and reduce the contents of the hernia sac. The surgeon sutures and ligates the sac, and assesses the floor of the canal. The mesh is then placed and sutured in a way that avoids excessive narrowing of the internal ring or trapping nerves during the repair. The surgeon ensures hemostasis and then closes the aponeurosis, fascia, and skin to complete the procedure. The surgeries in our study were conducted in accordance with the indications and contraindications of both procedures [13].

Laparoscopic and Lichtenstein mesh repairs have recently become popular as they provide a rapid return to normal activities with low recurrence rates [14]. Furthermore, a study found that mesh repair is superior to suture repair for preventing hernia recurrence, regardless of the size [15]. In this study, we aim to determine the relative frequency of factors associated with postoperative complications in hernia repair.

## Materials and methods

Study design

The study utilized a retrospective descriptive cohort design to examine patients who underwent hernia repair surgery at King Fahad Specialist Hospital in Qassim, Saudi Arabia, from 2020 to 2022.

Study area

The study was conducted at King Fahad Specialist Hospital, located in Qassim, Saudi Arabia. This hospital served as the primary setting for data collection.

Study population and sampling

The study population consisted of all consecutive patients who underwent hernia repair surgery during the specified period from 2020 to 2022. Patients were included if they met the following criteria: (a) underwent hernia repair surgery, and (b) had their surgery performed between 2020 and 2022. Patients were excluded if they had no history of hernia, had incomplete medical records, or had their hernia repair surgery performed before 2020 or after 2022.

Data collection

A questionnaire was developed as the research instrument to collect data from the patients. The questionnaire included items related to patients' biodata, demographics, relevant clinical features of hernia, established epidemiological risk factors, the type of intervention, and the type of complications. The questionnaire was designed to gather comprehensive information about the patients and their surgical procedures.

Validation of the research instrument (questionnaire)

The questionnaire underwent a validation process to ensure its reliability and validity. This process involved a pilot study in which a small sample of participants completed the questionnaire, and the data obtained were analyzed for internal consistency and clarity of the questionnaire items. Any necessary modifications or improvements to the questionnaire were made based on the pilot study results.

Data management and analysis

Data collected from the questionnaires was entered into a computer using the Microsoft Excel® package for efficient data management. The data were then exported to the SPSS software, version 23.0 (IBM Corp., Armonk, NY) for data analysis. Descriptive statistics, including frequencies and measures of centrality, were calculated to summarize the data. Inferential statistics were also employed to examine any significant associations or relationships between variables of interest.

Ethical considerations

Ethical approval was obtained from the Regional Research Ethics Committee, Qassim Province (approval no. H-04-Q-001) before initiating the study. Patient confidentiality and data protection were ensured throughout the study. Informed consent was obtained from the participants or their legal representatives, and personal identifiers were removed or anonymized during data analysis and reporting to maintain privacy and confidentiality.

## Results

In the current study, we were able to collect data from 274 patients with hernia. The majority of participants were male (79.9%, n = 219), while females accounted for 20.1%, n = 55, of the sample. In terms of body mass index (BMI), the distribution was as follows: (2.3%, n = 6) underweight, (30.2%, n = 80) normal weight, (37.7%, n = 100) overweight, and (29.8%, n = 79) obese. Regarding smoking status, 80.3% (n = 220) of participants reported not smoking, while 19.7% (n = 54) were smokers (Table 1).

**Table 1:** Demographic factors of the participants BMI, Body Mass Index

Demographic factors of the participants		Count	Column N %
Gender	Male	219	79.9%
	Female	55	20.1%
BMI	Underweight	6	2.3%
	Normal weight	80	30.2%
	Overweight	100	37.7%
	Obese	79	29.8%
Smoking	No	220	80.3%
	Yes	54	19.7%

Among the participants, 82.8% (n = 227) did not experience any complications, while 17.2% (n = 47) reported complications. The types of complications observed were as follows: seroma formation (2.9%, n = 8), wound infection (5.8%, n = 16), mesh infection (1.8%, n = 5), mesh extraction (1.5%, n = 4), wound hematoma (1.1%, n = 3), and pain (4.0%, n = 11) (Table 2).

**Table 2:** The prevalence and type of complications

The prevalence and type of complications		Count	Column N %
Complications	No	227	82.8%
	Yes	47	17.2%
Type of complications	No complications	227	82.8%
	Seroma formation	8	2.9%
	Wound infection	16	5.8%
	Mesh infection	5	1.8%
	Mesh extraction	4	1.5%
	Wound hematoma	3	1.1%
	Pain	11	4.0%

The distribution of hernia sites among the participants was as follows: abdominal wall/paraumbilical (30.5%, n = 83), umbilical (30.1%, n = 82), inguinal (18.8%, n = 51), below umbilicus (15.1%, n = 41), and above umbilicus (5.5%, n = 15). Bowel necrosis was reported in 5.5% (n = 15) of participants. The majority of admissions were elective (82.1%, n = 224) rather than emergency (17.9%, n = 49). Mesh use was reported in 78.9% (n = 213) of cases, while 9.6% (n = 26) did not receive mesh and 11.5% (n = 31) were unsure about its use. The technique employed for hernia repair was open in 73.9% (n = 201) of cases and laparoscopic in 26.1% (n = 71). A recurrence of hernia was observed in 19.7% (n = 54) of participants (Table 3).

**Table 3:** The characteristics of hernia

The characteristics of hernia		Count	Column N %
Site of hernia	Abdominal wall/paraumbilical	83	30.5%
	Umbilical	82	30.1%
	Inguinal	51	18.8%
	Below umbilicus	41	15.1%
	Above umbilicus	15	5.5%
Did you have bowel necrosis	No	258	94.5%
	Yes	15	5.5%
Mode of admission	Elective	224	82.1%
	Emergency	49	17.9%
Mesh use	No	26	9.6%
	Yes	213	78.9%
	Not known	31	11.5%
Technique	Open	201	73.9%
	Laparoscopic	71	26.1%
Is it Recurrent	No	220	80.3%
	Yes	54	19.7%

**Figure 1:**
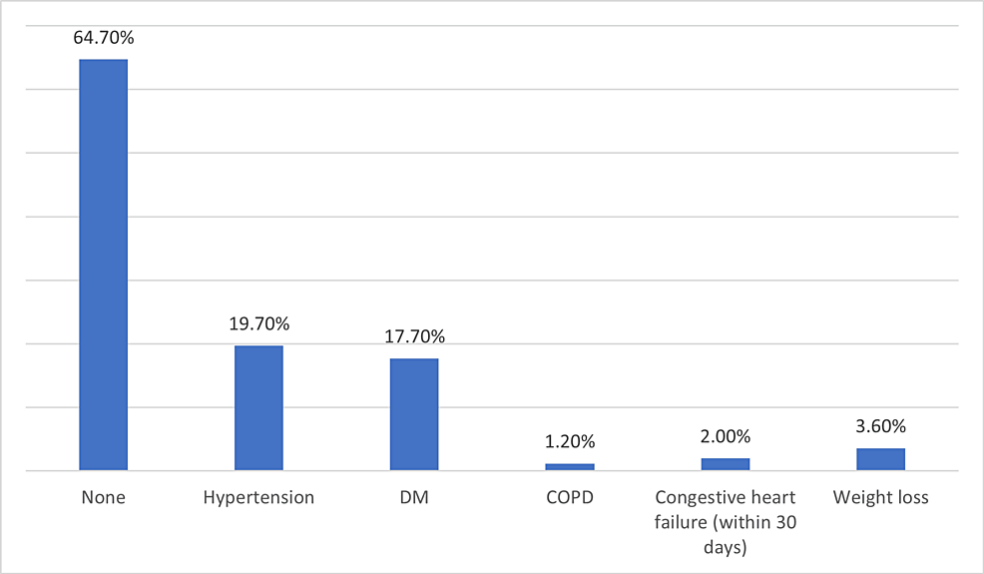
The prevalence of some comorbidities DM, Diabetes Mellitus; COPD, Chronic Obstructive Pulmonary Disease

Among the comorbidities assessed, the most prevalent was hypertension (19.7%, n = 54), followed by diabetes mellitus (17.7%, n = 48), weight loss (3.6%, n = 10), congestive heart failure within 30 days (2.0%, n = 5), chronic obstructive pulmonary disease (1.2%, n = 3), and no comorbidities (64.7%, n = 154) (Figure 1).

There were no significant associations between gender and the presence of complications. However, a significant association was found between BMI and complications (p < 0.001). Underweight participants had no complications, while the proportion of complications increased with higher BMI categories. The presence of comorbidities showed a significant association with complications (p = 0.031). Specifically, participants with comorbidities had a higher proportion of complications compared to those without comorbidities. Diabetes mellitus also showed a significant association with complications (p = 0.005), with a higher proportion of complications among participants with diabetes (Table 4).

**Table 4:** The relation between the presence of complications and demographic factors and comorbidities BMI, Body Mass Index; DM, Diabetes Mellitus; COPD, Chronic Obstructive Pulmonary Disease *Significant association with complication

The relation between the presence of complications and demographic factors and comorbidities		Complications				
		No		Yes		P-value
		Count	Row N %	Count	Row N %	
Gender	Male	184	84.0%	35	16.0%	0.305
	Female	43	78.2%	12	21.8%	
BMI	Underweight	6	100.0%	0	0.0%	0.001*
	Normal weight	69	86.3%	11	13.8%	
	Overweight	91	91.0%	9	9.0%	
	Obese	55	69.6%	24	30.4%	
Smoking	No	181	82.3%	39	17.7%	0.611
	Yes	46	85.2%	8	14.8%	
Comorbidities	Yes	87	77.0%	26	23.0%	0.031*
	No	140	87.0%	21	13.0%	
Hypertension	No	187	83.1%	38	16.9%	0.804
	Yes	40	81.6%	9	18.4%	
DM	No	197	85.7%	33	14.3%	0.005*
	Yes	30	68.2%	14	31.8%	
COPD	No	224	82.7%	47	17.3%	0.428
	Yes	3	100.0%	0	0.0%	
Congestive heart failure (within 30 days)	No	223	82.9%	46	17.1%	0.865
	Yes	4	80.0%	1	20.0%	
Weight loss	No	221	83.4%	44	16.6%	0.190
	Yes	6	66.7%	3	33.3%	

No significant associations were found between the prevalence of complications and the site of hernia, the presence of bowel necrosis, the mode of admission, mesh use, or the technique employed for hernia repair. However, a significant association was observed between the recurrence of hernia and the prevalence of complications (p = 0.007). Participants with recurrent hernias had a higher proportion of complications compared to those without a recurrence (Table 5).

**Table 5:** The relation between prevalence of complications and characteristics of hernia *Significant association with complication

The relation between the prevalence of complications and characteristics of hernia		Complications				
		No		Yes		
		Count	Row N %	Count	Row N %	
Site of hernia	Abdominal wall/paraumbilical	64	77.1%	19	22.9%	0.157
	Umbilical	68	82.9%	14	17.1%	
	Inguinal	48	94.1%	3	5.9%	
	Below umbilicus	34	82.9%	7	17.1%	
	Above umbilicus	12	80.0%	3	20.0%	
Did you have bowel necrosis	No	214	82.9%	44	17.1%	0.708
	Yes	13	86.7%	2	13.3%	
Mode of admission	Elective	185	82.6%	39	17.4%	0.856
	Emergency	41	83.7%	8	16.3%	
Mesh use	No	24	92.3%	2	7.7%	0.317
	Yes	176	82.6%	37	17.4%	
	Not know	24	77.4%	7	22.6%	
Technique	Open	166	82.6%	35	17.4%	0.922
	Laparoscopic	59	83.1%	12	16.9%	
Is it Recurrent	No	189	85.9%	31	14.1%	0.007*
	Yes	38	70.4%	16	29.6%	

The results of the regression analysis for the risk ratio of experiencing complications in hernia patients are summarized in Table 6. In terms of gender, females exhibited a 1.47 times higher risk of complications compared to males, although this finding was not statistically significant (p = 0.307). BMI categories showed varying associations, with obese individuals having a significantly higher risk (2.737 times) of complications compared to normal-weight individuals (p = 0.0132). Smoking status, comorbidities, and the presence of diabetes mellitus (DM) also demonstrated notable associations with complications. Specifically, smokers and individuals with comorbidities exhibited 0.807 times and 1.992 times higher risks, respectively, with the latter being statistically significant (p = 0.031). Patients with DM had a substantially elevated risk of complications (2.786 times) compared to those without DM (p = 0.005). The site of hernia was a significant predictor, with inguinal hernias associated with a remarkably lower risk (0.211 times) of complications compared to abdominal wall/paraumbilical hernias (p = 0.016). Additionally, the recurrence of hernia and the mode of admission were significant factors affecting complication risk, where recurrent cases and emergency admissions were associated with 2.567 times (p = 0.007) and 0.926 times (p = 0.856) higher risks, respectively. Other factors, including hypertension, COPD, congestive heart failure, weight loss, bowel necrosis, mesh use, and surgical technique, did not show statistically significant associations with the risk of complications in this hernia patient population (Table 6).

**Table 6:** Regression test of the risk ratio of having complications BMI, Body Mass Index; DM, Diabetes Mellitus; COPD, Chronic Obstructive Pulmonary Disease *Significant association with complication

Regression test of the risk ratio of having complications		Risk ration	95 % Confidence interval	P-value
Gender	Male	Reference		
	Female	1.47	0.70:3.05	0.307
BMI	Underweight	0.46	0.024:8.82	0.610
	Normal weight	Reference		
	Overweight	0.62	0.244: 1.580	0.317
	Obese	2.737	1.234: 6.07	0.0132*
Smoking	No	Reference		
	Yes	0.807	0.353: 1.844	0.611
Comorbidities	Yes	1.992	1.056: 3.756	0.031*
	No	Reference		
Hypertension	No	Reference		
	Yes	1.107	0.496: 2.471	0.804
DM	No	Reference		
	Yes	2.786	1.337: 5.802	0.005*
COPD	No	Reference		
	Yes	0.675	0.034: 13.289	0.428
Congestive heart failure (within 30 days)	No	Reference		
	Yes	1.212	0.132: 11.094	0.865
Weight loss	No	Reference		
	Yes	2.511	0.605: 10.423	0.190
Site of hernia	Abdominal wall/paraumbilical	Reference		
	Umbilical	0.694	0.321: 1.498	0.352
	Inguinal	0.211	0.058: 0.753	0.016*
	Below umbilicus	0.694	0.265: 1.814	0.455
	Above umbilicus	0.842	0.215: 3.297	0.805
Do you have bowel necrosis	No	Reference		
	Yes	0.748	0.163: 3.433	0.708
Mode of admission	elective	Reference		
	emergency	0.926	0.403: 2.128	0.856
Mesh use	No	Reference		
	Yes	2.522	0.571: 11.143	0.221
Technique	Open	Reference		
	Laparoscopic	0.965	0.469: 1.982	0.922
Is it Recurrent	No	Reference		
	Yes	2.567	1.278: 5.153	0.007*

## Discussion

The objective of this study was to ascertain the comparative occurrence of parameters linked to postoperative complications in hernia repair as well as to evaluate the prevalence of those difficulties. The study's results offer significant contributions to our understanding of the demographic variables and comorbidities that could influence the development of problems subsequent to hernia repair surgery.

Regarding the demographic variables, the findings of the study indicated that a significant proportion of the participants were of the male gender. This observation aligns with prior research that has documented a greater incidence of hernias in males in comparison to females [16-17]. Men are more likely to get hernias than women because of differences in their anatomy and physiology, such as having a wider inguinal canal and weaker abdominal wall components [16]. Nevertheless, the research conducted did not discover a statistically significant correlation between gender and the occurrence of problems. This finding indicates that gender might not exert a significant influence as a risk factor for postoperative complications in the context of hernia repair, which is similar to a previous study [18].

The study also evaluated the body mass index (BMI), which is a significant demographic variable. The results of the study indicated a statistically significant correlation between body mass index (BMI) and the occurrence of problems. Individuals classified as underweight did not report any difficulties; however, the incidence of complications exhibited an upward trend as the BMI categories increased. This finding aligns with prior studies that have demonstrated a correlation between obesity and a heightened susceptibility to problems in the context of hernia repair surgery [19]. There is a well-established correlation between obesity and several negative outcomes, including compromised wound healing, elevated infection rates, and heightened recurrence rates [20-21]. The results of this study underscore the significance of using BMI as a prognostic indicator for problems in individuals undergoing hernia repair surgery.

It is widely recognized that smoking poses a significant risk for postoperative problems in a range of surgical interventions, such as hernia repair [22]. Nevertheless, the present investigation could not identify a statistically significant correlation between smoking and the occurrence of problems. The absence of statistical significance could potentially be attributed to the limited sample size of people who self-reported engaging in smoking behavior, which accounted for 19.7% (n = 54) of the total population under study.

The presence of comorbidities has been identified as a substantial prognostic factor for postoperative complications in hernia repair surgery [23-24]. This study found a statistically significant relationship between the existence of comorbidities and the occurrence of problems. Individuals who presented with comorbidities exhibited a greater incidence of problems in comparison to those who did not have any comorbidities. Significant associations were observed between complications and diabetes mellitus (DM) among the comorbidities that were evaluated. The aforementioned discovery aligns with prior research that has established DM as a risk factor for wound infection, compromised wound healing, and increased rates of recurrence subsequent to hernia surgery [25]. In this investigation, it was shown that hypertension, which is a frequently occurring comorbidity, did not exhibit a statistically significant correlation with problems. Nevertheless, it is crucial to take into account that hypertension could potentially have an indirect impact on complications by influencing cardiovascular well-being and elevating the likelihood of perioperative problems.

The study's findings offer significant insights into the postoperative outcomes of hernia surgery by examining the frequency and nature of problems seen. The study found that the total incidence of problems was 17.2% (n = 47). Among these complications, seroma development and wound infection were the most frequently reported types. The results presented in this study align with prior research that has identified seroma development and wound infection as the most prevalent problems observed in hernia repair procedures [26-27]. The study findings suggest that the relatively low occurrence of problems can be related to several factors, such as the high level of proficiency exhibited by the surgical team, the utilization of acceptable surgical procedures, and the adherence to perioperative care standards. Nevertheless, it is crucial to acknowledge that the documented incidence can potentially underestimate the actual prevalence due to potential underreporting or oversight of certain difficulties throughout the duration of the follow-up period.

The study evaluated many characteristics of hernias, such as hernia location, presence of intestinal necrosis, method of admission, usage of mesh, repair technique, and recurrence. However, no significant relationships were found between these factors and the occurrence of problems. The present findings are consistent with other research that has documented varying relationships between these variables and the occurrence of postoperative problems in hernia surgery [28]. Nevertheless, it is crucial to take into account that the study's sample size and distribution of hernia features might have constrained the capacity to identify significant relationships. Additional investigation is necessary to go into these correlations in greater depth, necessitating larger sample sizes and encompassing a wider range of hernia features.

The findings of this study carry great significance in the field of clinical practice and the treatment of patients undergoing hernia repair surgery. The observed correlation between body mass index (BMI) and the occurrence of problems underscores the significance of providing preoperative counseling and optimizing care for individuals with elevated BMI. Obese patients may benefit from weight loss programs and increased vigilance in monitoring wound healing to mitigate the potential for consequences. The strong correlation between the existence of comorbidities, including diabetes mellitus (DM), and complications underscores the necessity of implementing a multidisciplinary approach and perioperative intervention for the management of these illnesses, with the aim of enhancing surgical outcomes. Furthermore, the significant occurrence of seroma formation and wound infection highlights the necessity of applying preventative measures, including precise surgical techniques, antibiotic prophylaxis, and suitable wound care protocols, in order to decrease the frequency of these complications.

Nevertheless, it is important to acknowledge that there exists a body of research that presents contradictory findings with regard to the correlation between specific parameters and postoperative problems in hernia repair procedures. For instance, the absence of a substantial correlation between gender and problems observed in this study aligns with findings from other research investigations [29]. Nevertheless, previous research has indicated a greater likelihood of problems among females [30]. The divergent results can be attributed to disparities in the demographics of the study participants, the magnitude of the sample sizes, and the methodology employed. Additional investigation is required to elucidate the impact of gender on postoperative problems in hernia repair.

The study demonstrates several notable qualities, such as its special concentration on a distinct cohort undergoing hernia repair surgery and its thorough evaluation of demographic variables, comorbidities, and hernia characteristics. The results of this study make a valuable addition to the current scholarly discourse surrounding the factors linked to postoperative problems in hernia repair procedures.

Limitation

It is crucial to acknowledge a number of constraints that must be taken into account when interpreting the findings. The research conducted utilized a sample size that was rather small, potentially resulting in low statistical power to identify significant relationships, particularly for less frequent complications and specific hernia characteristics. The study design's retrospective nature potentially generated biases related to selection and recall. Furthermore, it is worth noting that the study was carried out at a singular institution, thereby constraining the extent to which the results can be applied to different contexts.

## Conclusions

This research offers significant contributions by shedding light on the comparative occurrence of parameters linked to postoperative problems in hernia repair and the incidence of those difficulties. The results underscore the importance of demographic variables, such as body mass index (BMI) and the presence of comorbidities, in forecasting the likelihood of problems. The association between obesity and the coexistence of comorbidities, including diabetes mellitus, has been recognized as notable risk factors for problems in the surgical procedure of hernia repair. The high incidence of complications, such as seroma development and wound infection, emphasizes the importance of using preventative measures and optimizing perioperative care. The aforementioned findings possess the potential to contribute valuable insights to the process of clinical decision-making and perioperative care methods, hence enhancing the overall surgical outcomes in the context of hernia repair. Additional investigation is warranted to validate these correlations and investigate supplementary variables that could potentially contribute to postoperative problems in hernia surgery by utilizing larger sample sizes and standardized outcome measures.
